# *BclX*_*L*_ (*Bcl2l1*) gene therapy lessens retinal ganglion cell soma loss but not axonal degeneration after acute axonal injury

**DOI:** 10.1038/s41420-022-01111-4

**Published:** 2022-07-22

**Authors:** Olivia J. Marola, Sarah E. R. Yablonski, Peter G. Shrager, Robert W. Nickells, Richard T. Libby

**Affiliations:** 1 Department of Ophthalmology, Flaum Eye Institute, University of Rochester Medical Center, Rochester, NY USA; 2 Cell Biology of Disease Graduate Program, University of Rochester Medical Center, Rochester, NY USA; 3 The Center for Visual Sciences, University of Rochester, Rochester, NY USA; 4 Department of Neuroscience, University of Rochester, Rochester, NY USA; 5 Department of Ophthalmology and Visual Sciences, University of Wisconsin-Madison, Madison, WI USA; 6 Department of Biomedical Genetics, University of Rochester Medical Center, Rochester, NY USA

**Keywords:** Apoptosis, Cell death in the nervous system

Glaucoma, a leading cause of irreversible blindness, is characterized by loss of retinal ganglion cells (RGCs). In glaucoma, RGCs are thought to sustain axonal injury at the glial lamina [1]. This injury triggers molecularly distinct cell death pathways governing degeneration of the RGC soma and the distal axon. Much work has elucidated the mechanisms controlling degenerative processes in both RGC compartments [2]. In ocular hypertensive DBA/2J mice and after acute mechanical RGC axonal injury (controlled optic nerve crush, CONC), the apoptotic molecule BAX was shown to be required for degeneration of the soma, but not distal Wallerian degeneration of the axon [3]. In contrast, manipulation of molecules important for axonal degeneration (e.g. expression of *Wld*^*S*^) lessened death of the entire RGC in DBA/2J glaucoma [1]. Of note, after CONC (which allows independent analysis of the RGC somal and axonal compartments), *Wld*^*S*^ expression significantly delayed axonal degeneration but did not lessen RGC somal degeneration [4]—suggesting WLD^S^’s activity is restricted to the RGC axon. Taken together, these data suggest axon-localized degenerative pathways ultimately drive degeneration of both RGC compartments in glaucoma. In contrast, there is evidence that effectors originating from the soma are important in initiating axonal degeneration after neurodegenerative injury [5], suggesting that the factor(s) governing both somal and axonal degeneration in glaucoma may be initially triggered in the soma. Elucidating the inciting mechanism(s) driving both somal and axonal degeneration after glaucoma-relevant injury will be important in the development of neuroprotective therapies.

Recently, it was shown that overexpression of *BclX*_*L*_ protected the entire RGC in DBA/2J glaucoma [6]. BCLX_L_ inhibits BAX induction and is the principal pro-survival family member of the Bcl2 gene family expressed in RGCs [7]. *BclX*_*L*_ deletion significantly increased RGC death after CONC, suggesting BCLX_L_ activity protects RGCs after glaucoma-relevant injury [8]. BCLX_L_ was shown to localize to both somas and axons in dorsal root ganglion neurons [5]. Given this, it is possible that loss of BCLX_L_ activity from the RGC soma, axon, or from both compartments, drives RGC degeneration after glaucoma-relevant injury. Locating BCLX_L_’s protective effect will aid in understanding the role of somal and axonal contributions to RGC degeneration in glaucoma. Here, we utilize CONC to investigate the protective effect of *BclX*_*L*_ overexpression in the RGC soma and axon compartments independently.

To study the compartment-specific effects of *BclX*_*L*_ overexpression after CONC, *BclX*_*L*_ was overexpressed (*BclX*_*L*_^*AAV*^) in the retinas of C57BL/6J mice (aged 3–7 months) by bilateral intravitreal delivery of AAV2.2-Pgk-mCherry-BclX_L_ vector, performed as previously described [6]. Control animals (WT) were bilaterally intravitreally injected with volume-matched PBS. Mice were randomly selected to receive intravitreal AAV2.2-Pgk-mCherry-BclX_L_ or PBS. Mice were fed chow and water *ad libitum* and housed on a 12-hour light-to-dark cycle. All experiments were conducted in adherence with the Association for Research in Vision and Ophthalmology’s statement on the use of animals in ophthalmic and vision research and were approved by the University of Rochester’s University Committee on Animal Resources. A priori exclusionary criteria included abnormal eye phenotypes (e.g. shrunken eye, cataracts, displaced pupil, lens damage). CONC (performed as previously described [9]) was done no earlier than 28 days after intravitreal injection to allow for sufficient transduction. To determine gross physiological function of RGC somas, pattern electroretinography (PERG) was performed using the Celeris Diagnosys system according to manufacturer’s instructions. To assess physiological function of RGC axons, compound action potentials (CAPs) were recorded as previously described [4, 9] with peak amplitudes measured at 37 °C. Immunohistochemistry and imaging for retinal flat mounts and optic nerve longitudinal sections were performed as previously described [9] using antibodies against RBPMS (Genetex, GTX118619, 1:250), RFP (Chromotek, 5f8-100, 1:1000), cCASP3 (R&D, AF835, 1:1000), and Neurofilament (Millipore, AB5539SP, 1:1000). RBPMS+ cell counts and soma size measurements were performed using Image J. In all cases, experimenters were masked to experimental group and condition. Experimental groups had roughly equal numbers of males and females, were sex- and age-matched, and littermates were used wherever possible. Power analyses were performed a priori to determine appropriate sample sizes. Data are reported as mean ± standard error of the mean, and in all cases, data sets being compared had similar variances and met the assumptions of each statistical test used.

To determine the compartment-specific effect of *BclX*_*L*_ overexpression after mechanical axonal injury, CONC was performed on *BclX*_*L*_^*AAV*^ and WT control mice. Of note, as assessed by the percentage of mCherry+ RBPMS + cells, AAV2.2-Pgk-mCherry-BclX_L_ transduced ~76% of RGCs (Fig. 1A), consistent with previously published results [6]. Five days post-CONC, *BclX*_*L*_^*AAV*^ retinas had significantly fewer dying (cCASP3+) RGCs (Fig. 1B), and 14 days post-CONC, had significantly improved RGC survival compared to WT controls (Fig. 1C). Therefore, consistent with previous reports [6, 10], *BclX*_*L*_ overexpression improved RGC somal survival after axonal injury. These data suggest loss of BCLX_L_ activity in the soma contributes to RGC somal degeneration in glaucoma and could also possibly contribute to degeneration of the axonal compartment.

**Fig. 1:**
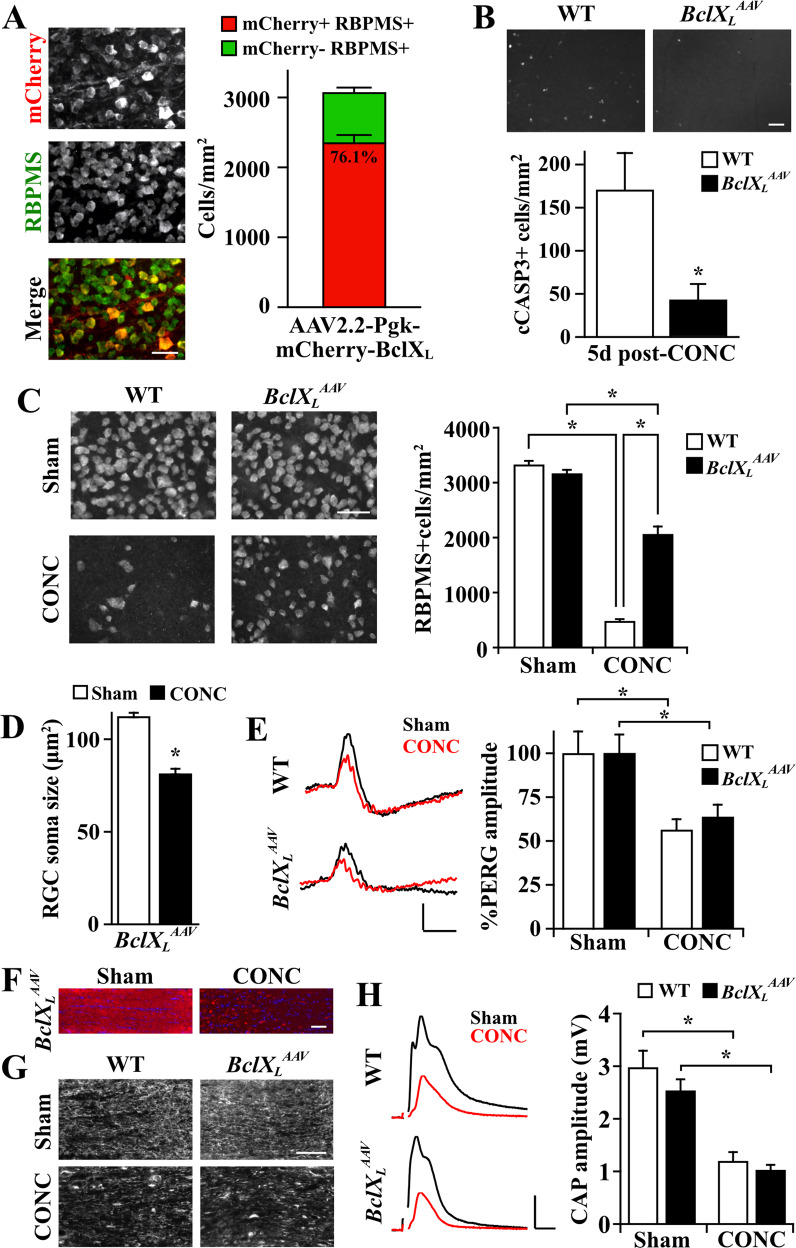
*BclX*_*L*_ overexpression improved RGC somal survival but not axonal degeneration after CONC. **A** Transduction efficiency of AAV2.2-Pgk-mCherry-BclX_L_ in RGCs as assessed by the percentage of mCherry+ (red) RGCs (RBPMS + cells, green) depicted in retinal flat mounts. On average, 76.1 ± 1.2% of RGCs were colabeled with mCherry. *n* = 4. Scale bar, 50 µm. **B** WT (*n* = 5) and *BclX*_*L*_^*AAV*^ (*n* = 6) retinal flat mounts and quantification of cleaved caspase 3 (cCASP3)+ cells 5 days post-CONC. *BclX*_*L*_^*AAV*^ retinas had 74.9 ± 10.5% fewer cCASP3+ cells compared to WT controls. *P = 0.030, two-tailed *t-*test. Scale bar, 50 µm. **C** WT and *BclX*_*L*_^*AAV*^ retinal flat mounts and quantification of RGCs (RBPMS + cells) 14 days post-CONC. Both WT and *BclX*_*L*_^*AAV*^ retinas had significant RBPMS + cell loss after CONC compared to Sham controls (85.4 ± 0.8% and 34.8 ± 4.2% loss respectively, *P < 0.001). However, *BclX*_*L*_^*AAV*^ retinas had 59.2 ± 4.9% improved RGC survival after CONC compared to WT controls (*P < 0.001). *n* = 5, two-way ANOVA, Holm-Sidak’s post hoc test. Scale bar, 50 µm. **D** Quantification of RBPMS + RGC soma size from *BclX*_*L*_^*AAV*^ retinas 14 days after Sham and CONC. After CONC, surviving RGCs from *BclX*_*L*_^*AAV*^ retinas were 27.5 ± 2.2% smaller compared to Sham controls. *n* = 5, *P < 0.001, two-tailed *t-*test. **E** Representative PERG traces and quantification of PERG amplitudes from WT and *BclX*_*L*_^*AAV*^ eyes 14 days post-Sham (*n* = 17, 18, respectively) and CONC (*n* = 18, 17, respectively). WT and *BclX*_*L*_^*AAV*^ eyes had significant reductions in PERG amplitude after CONC relative to Sham (43.5 ± 5.8% and 36.2 ± 6.6% reductions respectively, *P < 0.05). *BclX*_*L*_^*AAV*^ eyes did not have improved PERG amplitudes after CONC compared to WT controls (*P* = 0.816). Two-way ANOVA, Holm-Sidak’s post hoc test. Scale bar: X: 100 ms, Y: 5µV. **F** Longitudinal *BclX*_*L*_^*AAV*^ optic nerve sections 5 days post-Sham and CONC. Sham *BclX*_*L*_^*AAV*^ optic nerves had notable axonal mCherry labeling, which was markedly “beaded” and lost post-CONC. *n* = 4. Scale bar, 50 µm. **G** Longitudinal WT and *BclX*_*L*_^*AAV*^ optic nerve sections 5 days post-Sham and CONC immunoassayed for neurofilament-H. *BclX*_*L*_^*AAV*^ optic nerves had similar histological signs of degeneration after CONC compared to WT controls. *n* = 4. Scale bar, 50 µm. **H** Representative CAP traces and quantification of CAP amplitudes from WT and *BclX*_*L*_^*AAV*^ optic nerves 5 days post-Sham and CONC. Both WT and *BclX*_*L*_^*AAV*^ optic nerves had significantly decreased CAP amplitudes after CONC compared to Sham controls (59.7 ± 5.6% and 59.3 ± 3.7% amplitude reductions respectively, *P < 0.001). After CONC, *BclX*_*L*_^*AAV*^ optic nerves did not have improved CAP amplitudes compared to WT controls (*P* = 0.582)*. n* = 5, two-way ANOVA, Holm-Sidak’s post hoc test. Scale bar: X: 1 ms, Y: 1 mV. All numerical data are reported as mean ± standard error of the mean. For graphs, bars represent the mean, and error bars represent standard error of the mean.

Strikingly, despite improved somal survival in *BclX*_*L*_^*AAV*^ retinas, surviving *BclX*_*L*_^*AAV*^ RGC somas were significantly shrunken 14 days post-CONC compared to Sham controls (Fig. 1D), suggesting injury or metabolic stress [11, 12]. This somal shrinkage was also observed in *Bax* deficient RGCs after CONC [13]. In addition, *BclX*_*L*_ overexpression was not sufficient to prevent a decrease in PERG amplitude (which is thought to be reflective of RGC activity [14]) 14 days after CONC (Fig. 1E). Thus, while *BclX*_*L*_ overexpression improved RGC soma survival after CONC, RGC somas did not appear to retain normal function. These data imply the separable nature of the mechanisms governing RGC somal survival and retention of physiological function.

Given that *BclX*_*L*_ overexpression protected RGC axons and somas in a model of ocular hypertension [6], it remained important to distinguish whether somal BCLX_L_ confers protection to the RGC axon, or if axonal BCLX_L_ affords this protection. To investigate this, axonal degeneration of *BclX*_*L*_^*AAV*^ and WT optic nerves was assessed after CONC. Of note, the BCLX_L_ fusion protein (mCherry) prominently co-localized to RGC axons in the optic nerve (Fig. 1F), as was shown previously [6]. Axonal health was assessed histologically (labeling for neurofilament-H) and electrophysiologically by measuring CAPs. *BclX*_*L*_ overexpression did not lessen histological hallmarks of RGC axonal degeneration (Fig. 1G), nor prevent CAP amplitude decline after CONC (Fig. 1H). Thus, *BclX*_*L*_ overexpression did not appear to elicit neuroprotective effects by acting in the RGC axon after glaucoma-relevant injury.

Taken together, these data suggest that the detrimental effect of BCLX_L_ loss may be localized to the soma in the context of glaucomatous injury. This implicates the importance of degenerative mechanisms initiated in the RGC soma in ultimately driving death of the entire RGC. Future work should elucidate the mechanisms by which loss of somal BCLX_L_ activity initiates axonal degenerative activity to further uncover the earliest drivers of glaucomatous neurodegeneration.

## Supplementary information

Reproducibility Checklist

## Data Availability

The datasets used in the current study are available from the corresponding author on reasonable request.
